# Supplementary material for: Involvement of M1-Activated Macrophages and Perforin/Granulysin Expressing Lymphocytes in IgA Vasculitis Nephritis
Source: Int J Mol Sci. 2024 Feb 13;25(4):2253. doi: 10.3390/ijms25042253 (PMC10889255; doi:10.3390/ijms25042253)
Supplement: Supplementary file 1 [file ijms-25-02253-s001.zip › Table 3 Supplementary File.pdf]

Table 3. Antibodies used in the study.

Target	Catalog number [Clone]	Company	Dilution
**Primary antibodies**			
CD68	ab199000 [KP1 + C68/684], mouse IgG1	Abcam, Cambridge, UK	1:100
CD68	ab192847 [SP251], rabbit	Abcam, Cambridge, UK	1:100
iNOS	ab115819 [SP126], rabbit	Abcam, Cambridge, UK	1:100
Arginase-1	ab 212522 [ARG1/1125] mouse IgG3	Abcam, Cambridge, UK	1:25
CD3	ab21703 [SP7], rabbit	Abcam, Cambridge, UK	prediluted
NCAM (CD56)	ab204446, polyclonal rabbit	Abcam, Cambridge, UK	1:100
NKp44	MAB2249 [253422] mouse IgG2b	R&D Systems, Minneapolis, Minnesota, USA	1:20
Perforin	556434 [δG9] mouse IgG2b	BD Biosciences, San Diego, California, USA	1:100
Granulysin	D-185-3 [RC8] mouse IgG1	MBL International, Woburn, MA, USA	1:100
**Isotype controls**			
Mouse IgG1	BD554121 [MOPC-21]	BD Bioscience, San Diego, California, USA	1:100
Mouse IgG2a	14-4724-82 [eBM2a]	Thermo Fisher Scientific, Waltham, Massachusetts, USA	1:100
Mouse IgG2b	BD557351 [MPC-11]	BD Biosciences, San Diego, California, USA	1:100
Mouse IgG3	14-4742-82 [B10]	Thermo Fisher Scientific, Waltham, Massachusetts, USA	1:100
Rabbit IgG	ab37415 [IgG]	Abcam, Cambridge, UK	1:200
**Secondary antibodies**			
Anti-mouse IgG Alexa Fluor 488	A-11001 [goat IgG]	Thermo Fisher Scientific, Waltham, Massachusetts, USA	1:300
Anti-rabbit IgG Alexa Fluor 594	A-11037 [goat IgG]	Thermo Fisher Scientific, Waltham, Massachusetts, USA	1:500
Anti-mouse IgG Alexa Fluor 594	A 21203 [donkey IgG]	Thermo Fisher Scientific, Waltham, Massachusetts, USA	1:300
Anti-rabbit IgG Alexa Fluor 488	A-21206 [donkey IgG]	Thermo Fisher Scientific, Waltham, Massachusetts, USA	1:300

CD, Cluster of differentiation; IgG, Immunoglobulin G; iNOS, inducible Nitric Oxide Synthase; NCAM 1, Neural Cell Adhesion Molecule 1; NK, Natural Killer; NKp44, Natural Killer protein 44.
