# Supplementary figures and images for: Involvement of M1-Activated Macrophages and Perforin/Granulysin Expressing Lymphocytes in IgA Vasculitis Nephritis
Source: Int J Mol Sci. 2024 Feb 13;25(4):2253. doi: 10.3390/ijms25042253 (PMC10889255; doi:10.3390/ijms25042253)

## Slide 1
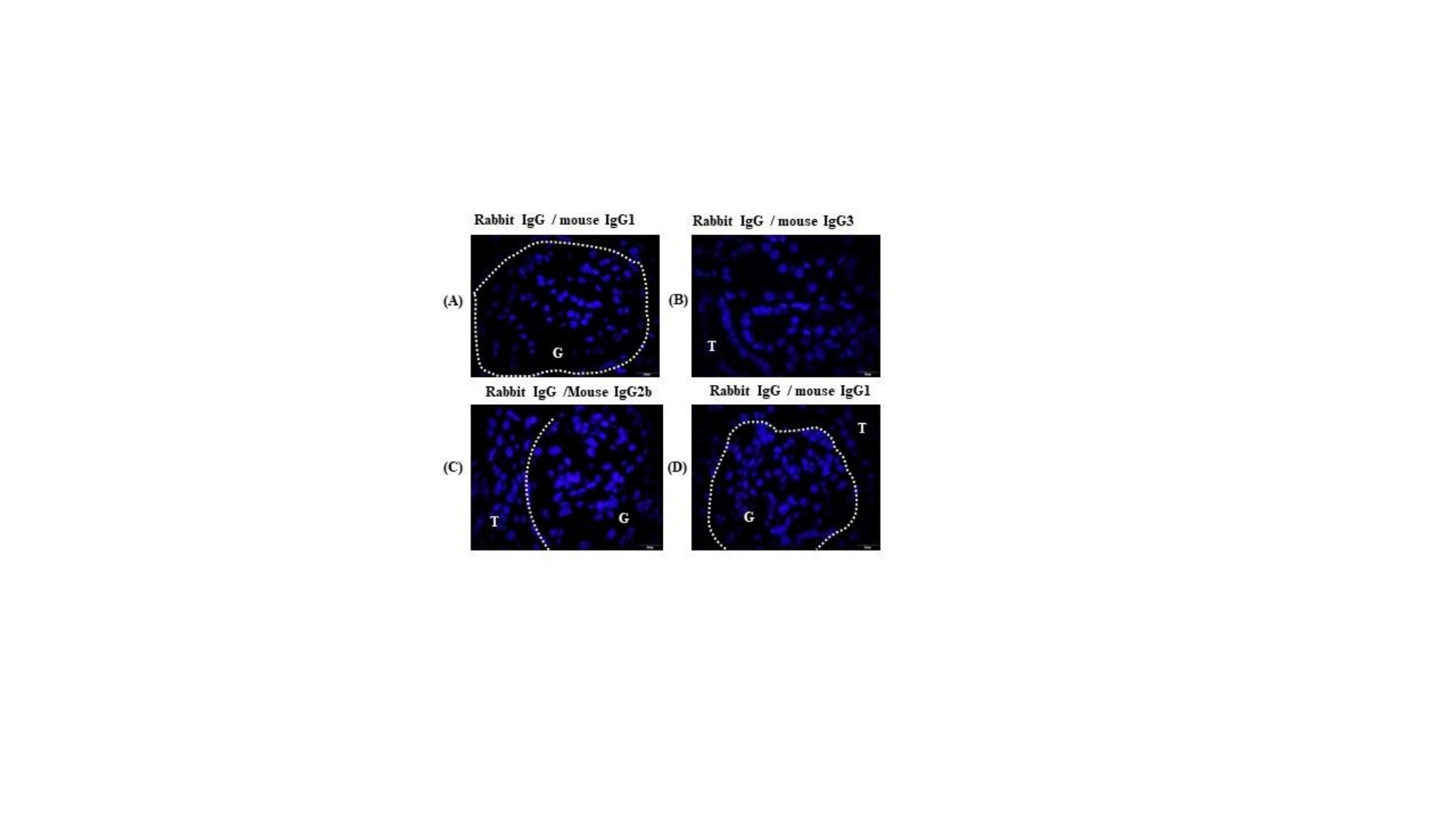

Supplement: Supplementary file 1 [file ijms-25-02253-s001.zip › Figure 5 Supplementary file.pptx]
